# Molecular signatures that correlate with induction of lens regeneration in newts: lessons from proteomic analysis

**DOI:** 10.1186/s40246-014-0022-y

**Published:** 2014-12-11

**Authors:** Konstantinos Sousounis, Rital Bhavsar, Mario Looso, Marcus Krüger, Jessica Beebe, Thomas Braun, Panagiotis A Tsonis

**Affiliations:** Department of Biology and Center for Tissue Regeneration and Engineering at Dayton, University of Dayton, 300 College Park, Dayton, OH 45469 USA; Department of Cardiac Development and Remodeling, Max-Planck-Institute for Heart and Lung Research, Ludwigstrasse 43, 61231 Bad Nauheim, Germany

**Keywords:** Regeneration, Lens, Newt, Proteomics, Gene expression, Regeneration program

## Abstract

**Background:**

Amphibians have the remarkable ability to regenerate missing body parts. After complete removal of the eye lens, the dorsal but not the ventral iris will transdifferentiate to regenerate an exact replica of the lost lens. We used reverse-phase nano-liquid chromatography followed by mass spectrometry to detect protein concentrations in dorsal and ventral iris 0, 4, and 8 days post-lentectomy. We performed gene expression comparisons between regeneration and intact timepoints as well as between dorsal and ventral iris.

**Results:**

Our analysis revealed gene expression patterns associated with the ability of the dorsal iris for transdifferentiation and lens regeneration. Proteins regulating gene expression and various metabolic processes were enriched in regeneration timepoints. Proteins involved in extracellular matrix, gene expression, and DNA-associated functions like DNA repair formed a regeneration-related protein network and were all up-regulated in the dorsal iris. In addition, we investigated protein concentrations in cultured dorsal (transdifferentiation-competent) and ventral (transdifferentiation-incompetent) iris pigmented epithelial (IPE) cells. Our comparative analysis revealed that the ability of dorsal IPE cells to keep memory of their tissue of origin and transdifferentiation is associated with the expression of proteins that specify the dorso-ventral axis of the eye as well as with proteins found highly expressed in regeneration timepoints, especially 8 days post-lentectomy.

**Conclusions:**

The study deepens our understanding in the mechanism of regeneration by providing protein networks and pathways that participate in the process.

**Electronic supplementary material:**

The online version of this article (doi:10.1186/s40246-014-0022-y) contains supplementary material, which is available to authorized users.

## Background

Several amphibian species own the ability to regenerate multiple different organs during adulthood making them excellent models to study the molecular mechanisms of tissue regeneration. Although regulation of regeneration might diverge among tetrapods, a deeper understanding of regenerative processes in amphibians will provide valuable clues for organ repair and regeneration in other organisms such as mammals [1,2].

Regeneration of the eye lens in newts provides a superb model to study regeneration. After surgical removal of the lens, the whole organ regenerates by transdifferentiation of dorsal iris pigmented epithelial (IPE) cells. Interestingly, the lens is never regenerated from the ventral iris, which provides a number of experimental options [3,4]. Most importantly, gene expression differences between the dorsal and the ventral part of the iris can be identified to unravel the molecular program enabling regeneration. Similarly, changes in the expression profile between the iris while the lens is intact and during lens regeneration allow characterization of regulatory pathways initiating regeneration. In addition, dorsal IPE cells retain their ability to form a lens after *in vitro* culturing, aggregation, and orthotopic transplantation or implantation into 3-D collagen lattices while ventral IPE aggregates fail to do so. Hence, gene expression profiles of cultured dorsal and ventral IPE cells might provide additional insights into lens regeneration [5].

Recently, the first newt transcriptome was assembled, and RNA sequencing of newt iris has been used to study differences in gene expression between the dorsal and ventral iris. Analysis of gene expression identified genes exclusively expressed in either the dorsal or ventral iris as well as genes expressed in a gradient along the dorsoventral axis of the iris during lens regeneration [6,7]. In another study, custom newt microarrays were used to detect 467 genes that were differentially expressed during lens regeneration [8]. Although these studies provided essential information about the expression of genes during newt lens regeneration, protein data were missing so far. Mass-spectrometry-based protein analysis closes this gap providing information about changes in protein concentrations and potential post-transcriptional regulation during lens regeneration. Here, we computed the newt proteome from the assembled transcriptome and performed liquid chromatography followed by tandem mass spectrometry (LC-MS/MS) to identify proteins differentially expressed between dorsal and ventral iris as well as between regenerating and intact iris. We chose to use dorsal and ventral iris 4 and 8 days post-lentectomy (dpl) since during these timepoints iris cells re-enter the cell cycle and transdifferentiate. We then compared the expression data with the previously reported gene expression data at the mRNA level collected at the same timepoints. In addition, we performed LC-MS/MS with samples collected from *in-vitro*-cultured dorsal and ventral IPE cells. We compared the expression profiles between the *in vitro* and *in vivo* experiments. Lastly, we compared available high-throughput mRNA and protein expression data obtained from amphibian organ model systems undergoing regeneration, identifying a common regeneration program.

## Results and Discussion

### LC-MS/MS identifies novel newt proteins

**Figure 1:**
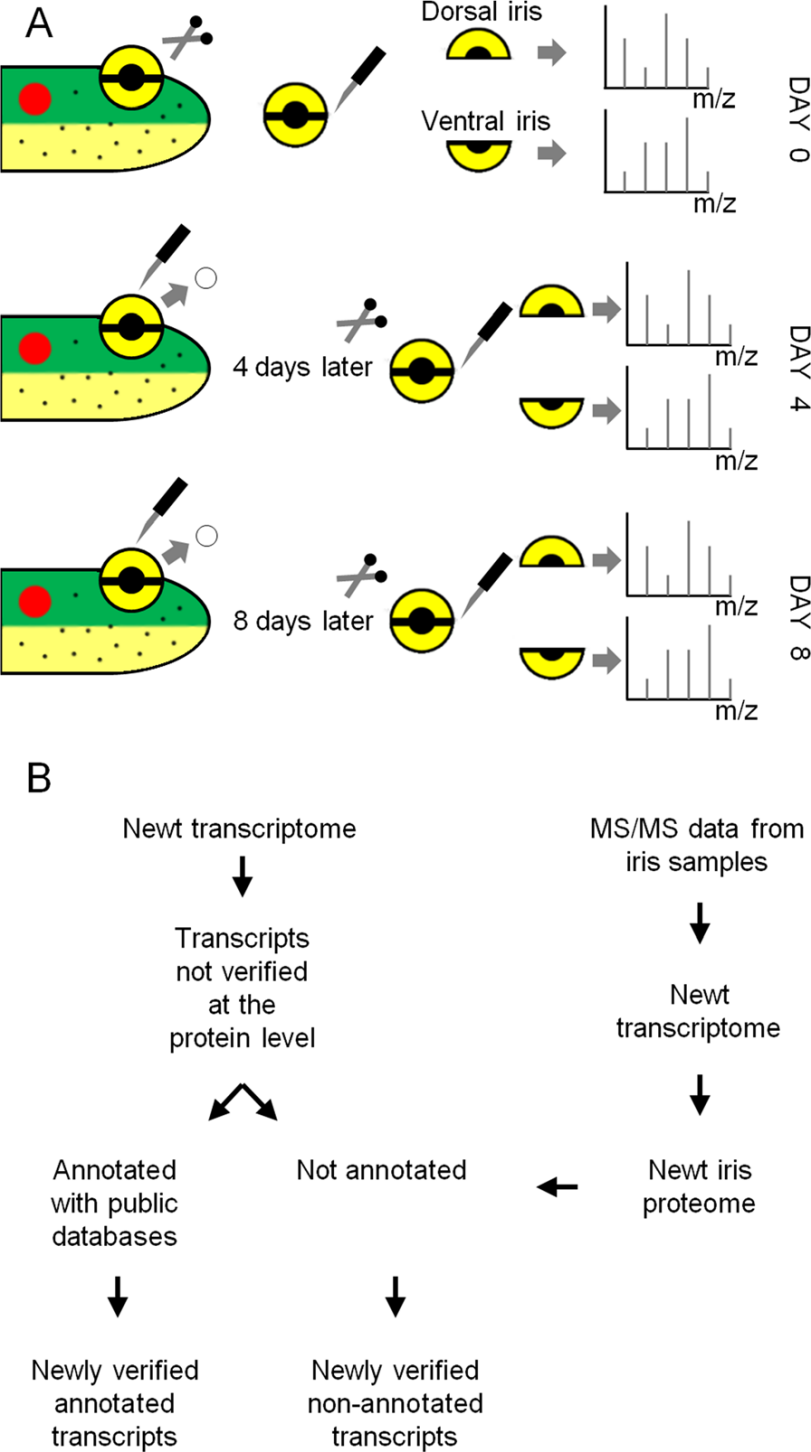
**Overview of the experimental procedure. (A)** Sample collection at 0, 4, and 8 days post-lentectomy. Lenses were surgically removed from the newt’s eyes and iris rings were split into dorsal-ventral halves before subject to LC-MS/MS. **(B)** Comparison between newly obtained data and previously described newt transcriptome results. Data were used to validate annotated transcripts at the protein level and identify non-annotated proteins that are probably unique to newts or amphibians.

Lenses were removed from newt eyes in order to initiate the regeneration process at the dorsal iris. Dorsal and ventral iris samples were collected at 4 and 8 dpl as well as from intact tissue (day 0). At 4 dpl, dorsal and ventral iris cells re-enter the cell cycle while at 8 dpl only dorsal iris cells initiate dedifferentiation. The iris samples were prepared, and LC-MS/MS was performed in order to investigate changes in protein concentrations (Figure 1A). The newly assembled newt transcriptome was used for peptide identification [7]. LC-MS/MS identified 8,167 different proteins. These proteins were uniquely annotated to 4,734 different human proteins (e-value < 1E-10). Direct comparisons with proteins found in previous proteomic studies in newts revealed that 701 of these annotated proteins have not been detected in newts before [7]. Our dataset also includes 143 proteins lacking annotations in other species, which raises the possibility that they are unique to newts. These data are summarized as a whole and per sample in Table 1 (Workflow: Figure 1B).

**Table 1:** **Number of annotated and non-annotated proteins found by LC-MS/MS**

		**Day 0**		**Day 4**		**Day 8**	
	**Total** ^**a**^	**Dorsal**	**Ventral**	**Dorsal**	**Ventral**	**Dorsal**	**Ventral**
Proteins expressed	8,167	3,454	4,899	4,082	3,616	4,474	5,374
New verified proteins	1,479	248	604	361	285	436	682
Human proteins expressed	4,734	2,638	3,425	2,997	2,705	3,269	3,621
New verified human proteins	701	150	333	199	159	242	353
New verified newt proteins	143	13	37	29	30	28	54

Values are given per sample and for all experiments together.

^a^non-redundant.

### Protein expression patterns during regeneration

**Figure 2:**
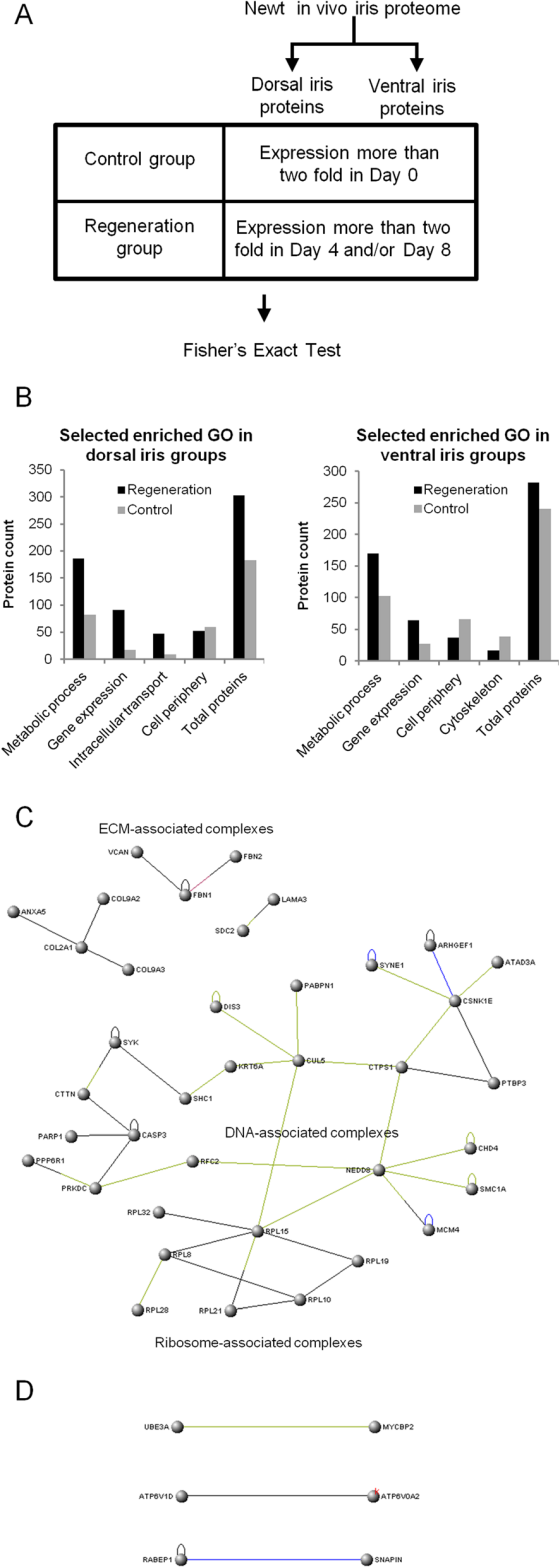
**Comparisons between regenerating and control samples. (A)** Workflow for selecting control and regeneration groups for Fisher’s exact test. **(B)** Selected enriched gene ontology (GO) terms (FDR < 0.05) in regeneration or control groups for dorsal and ventral samples. *Bars* indicate the number of proteins in each group. **(C)** Network analysis of proteins expressed at higher levels in regenerating and dorsal samples. **(D)** Network analysis of proteins expressed at higher levels in regenerating and ventral samples. **(C,** **D)** Connections between nodes indicate protein-protein interaction. Only proteins that showed at least one interaction with another protein of the same group were displayed.

The primary interest of our study was to identify proteins that might play a role during tissue regeneration. To achieve this goal, we compared proteins that were present at 4 and 8 dpl (both dorsal and ventral iris) and were expressed at least twofold higher than proteins in the intact iris at day 0 (regeneration group). Likewise, we examined proteins that were down-regulated (at least twofold) during regeneration (control group). To investigate the trends that our analysis yielded, we used the gene ontology (GO) terms of the annotated proteins and we performed enrichment analysis using Fisher’s exact test corrected for multiple selections between the regeneration and control groups (false discovery rate, FDR < 0.05; Figure 2A). Metabolic process and gene expression were some of the GO terms enriched during regeneration in both dorsal and ventral iris samples while the GO term “cell periphery” was enriched in the control samples (Figure 2B and Additional file 1). Tables 2 and 3 (for dorsal iris) and Tables 4 and 5 (for ventral iris) list the genes reflecting the GO term enrichments for the regeneration group. These genes determine cellular functions in response to stress and reactive oxygen species. They are also involved in the regulation of translation, RNA maturation, and immune responses. Genes that were induced in the iris post-lentectomy can be grouped per function as follows:

**Table 2:** **Genes with GO term related to gene expression that are up-regulated in the dorsal iris during regeneration versus control**

**GO:0010467; gene expression and parental GO**							
**Dorsal regeneration**						**Dorsal control**	
AARS	ENDOU	KHSRP	RPL10A	RPLP2	SNRNP70	AHCYL2	GJA1
ANK3	ETF1	KIAA1967	RPL13	RPS2	SOAT1	BACE2	KANK2
BUD31	FUBP1	LUC7L3	RPL13A	RPS23	SPEN	CAV1	KRT17
BZW1	GARS	METAP1	RPL18	RPS24	SSB	CCDC88C	MPP6
C3	GCN1L1	MTA2	RPL18A	RPS28	SUGP2	CRK	NEO1
CSDE1	GLG1	MTOR	RPL19	RPS4X	SUPT16H	EEF1A1	PSMB9
DAD1	HBS1L	PABPC1	RPL21	RPS5	TARS	EIF4H	PTRF
DDX17	HCK	PES1	RPL26	RPS6	TMED2	FHL2	RBCK1
DPM1	HMOX1	POLR2A	RPL27A	RPS8	U2AF1L4	FKBP9	STAT5B
EEF1D	HNRNPM	POLR2E	RPL28	RPS9	UBE2I		
EEF1G	HNRNPU	PPP2R5C	RPL3	SERBP1	VARS		
EEF2	HSPA8	PRMT5	RPL35	SLTM	WDR77		
EIF3B	ILF3	PRPF8	RPL4	SMARCA5	WFS1		
EIF4G2	IPO9	PTBP3	RPL5	SMC1A	YARS		
EIF5A	KHDRBS1	RPL10	RPL8	SND1	YBX1		
EIF5B

**Table 3:** **Genes with GO term related to metabolic process that are up-regulated in the dorsal iris during regeneration versus control**

**GO:0008152; metabolic process and parental GO**							
**Dorsal regeneration**				**Dorsal control**			
AASS	CYB5B	HSD17B13	PPID	ABAT	CP	ITPR2	PTPRA
ACTN1	CYBA	HSP90AB1	PPP3CC	ABCB7	CTSS	LANCL1	PTPRD
AFG3L2	CYBB	HSP90B1	PSMD1	ACAD10	CYP2A6	LTA4H	RAB27B
AKAP8L	DDX5	HSPA5	PTPRC	ACOT2	DCN	MAOA	RAB7A
ALDH16A1	DNAJA1	HSPA9	RAD23A	AGL	DCTN1	MINPP1	RECK
ALDH18A1	DNPEP	IRG1	RASA4	AK4	DHRS2	MRI1	SDHAF2
ALDOC	DPP3	ITGB2	RECQL	AKR1C4	ECHS1	MYH11	SDHD
ALOX5AP	DSP	ITPR2	RHO	ALDH3B1	EHD2	MYO5A	SH3GLB1
APMAP	DUSP11	LMNA	SCD5	ANXA1	ENTPD2	NEU3	SPTBN1
APOA1	ELOVL1	MAP3K15	SDHC	AOC3	FABP3	NPR3	SULT1B1
ASS1	ENPP4	MARCKS	SDR16C5	APPL2	GAPVD1	NRP1	SULT1C2
ATP2A2	ENTPD8	MPO	SERPINB10	ATP13A5	GGT5	PARG	TPM1
ATP6V0D1	EPX	MYO18A	SLC25A12	CDC42BPB	GRHPR	PGM5	TPP1
ATP6V1A	ERO1L	NAAA	SLC9A3R1	CECR1	GSTZ1	PIPOX	TUBB3
BIN1	F13A1	NAT8B	SQRDL	CLPX	HIBCH	PPIC	YWHAG
CANX	FAM213A	NCF1C	TIMM50				
CFB	FN1	NCF2	TNFAIP8				
CHIA	GNS	NUP93	TOP1				
COL3A1	GPD1L	PCCA	UBLCP1				
COX7A2L	GSTP1	PDPR	UQCRB				
CPT1A	H6PD	PFKP	USP5				
CTSL1	HK1	PGD	VRK1				
CUL3	HM13

**Table 4:** **Genes with GO term related to gene expression that are up-regulated in the ventral iris during regeneration versus control**

**GO:0010467; gene expression and parental GO**							
**Ventral regeneration**						**Ventral control**	
AARSD1	EIF3J	PFDN5	RPL27A	RQCD1	THBS1	CAT	ILK
ATP6AP2	ENDOU	POLR1C	RPL3	SEC11A	UBTF	CD44	KANK2
BZW1	GALNT12	PRKCI	RPL38	SMARCA5	VARS	CDH13	KRT17
CARS	HBS1L	PRMT5	RPL4	SNF8	VIPAS39	CHMP1A	PRKCA
CSDE1	HMOX1	PRPF8	RPL5	SNRPE	WARS	COG2	PRKDC
DDX39A	HSPA8	PSME3	RPS13	SOAT1	WDR61	COL4A6	PURA
DNAJC2	KDM1A	RCL1	RPS15	SPEN	WTAP	CTNNB1	RBCK1
EBNA1BP2	MARS	RPL10	RPS17	SRSF12	YBX1	DEK	SBDS
EEF1B2	METAP1	RPL13	RPS23	SSR4	YLPM1	DMD	SEC31A
EEF1D	NPC1	RPL17	RPS27	SUPT6H		EXOSC7	UGGT2
EEF1G	PABPC1	RPL18A	RPS8	TGFB1		GJA1	VPS36

**Table 5:** **Genes with GO term related to metabolic process that are up-regulated in the ventral iris during regeneration versus control**

**GO:0008152; metabolic process and parental GO**							
**Ventral regeneration**				**Ventral control**			
ABCF2	DCN	LARP1	PRRC1	ABCB5	ECHDC2	NIT2	SLC22A2
ACSBG2	DDX5	LYN	PRSS16	ADH4	EHD2	OXCT1	SLC9A3R2
ACY1	DGAT1	LYZ	PRTN3	AGL	F8	PI4KA	SOD1
ADH1B	DNAJB11	MCM5	PTPN6	AK4	FAM162A	PLCG1	SULT1B1
ALDH18A1	DNM2	MCM6	PTPRC	ALG11	FBN1	PNP	SULT1C2
ALDH1A3	ECI2	MDN1	PZP	ATAD1	GALE	PNPO	SYTL2
ALOX5	ECM1	MOB1B	RCC1	ATG7	GLUL	PRKAB1	TGM1
ALPL	ENPP4	MPDU1	RECQL	C6orf130	GMPR2	PRKAR2B	TPP1
ARAP1	EPX	MPO	RFC5	CAPN5	GNA14	PTGR1	TTLL12
ASS1	F13A1	MYO18A	RNF213	CDIPT	GNAI1	PTK2	TUBB3
ATP2A1	FAM213A	MYO5A	RNLS	CECR1	GRHPR	PTPLAD1	VCP
ATP6V0D1	FASN	NAAA	SDR16C5	CLYBL	HAGH	PTPRD	XPNPEP1
ATP6V1F	FBP1	NADKD1	SERPINB10	COPS3	HIST1H2AG	PYGB	
C3	FEN1	NCF1C	SERPINB6	CP	HMOX2	RAB27B	
CFB	FKBP4	NCF2	SGPP1	CTSS	KLC4	RAB3D	
CHIA	FN1	NCKAP1L	SLC25A40	CUL5	LANCL1	RAB7A	
CKM	GBF1	NMT2	SMPD3	CYP2A13	MARCKS	RABGAP1	
CNEP1R1	GCAT	NRAS	SQSTM1	CYP2J2	MTCH1	RGN	
COX5B	GCLM	NUP155	SYK	DCTN1	MT-CYB	RTN4IP1	
CP	GLB1	NUP210	TBC1D9B	DLG1	MYH11	SEPHS1	
CTSA	GLUL	P2RX4	TOP1	DMGDH	NDUFB4	SERPINI1	
CTSL1	HECTD1	PAFAH1B2	UNC45A				
CUL2	HIST2H2AB	PGD	USP24				
CYBB	IDE	PLD3	VWA8				
CYP4F22	IRG1	PNP	WBSCR22				
CYP7B1	ITGB2

- Gene expression

Elongation factor 1-delta (EEF1D), elongation factor 1-gamma (EEF1G), valine-tRNA ligase, and ribosomal proteins RPL10, RPL13, RPL18A, RPL27A, RPL3, RPL4, RPL5, RPS23, and RPS8 are all known for their role in the translation of proteins. RNA-processing proteins include poly(U)-specific endoribonuclease (ENDOU), nuclease-sensitive element-binding protein 1 (YBX1), which is also implicated in cell proliferation [9], pre-mRNA-processing-splicing factor 8 (PRPF8), and probable ATP-dependent RNA helicase DDX5. In addition, two members of the ribonucleoprotein complex (ARC), polyadenylate-binding protein 1 (PABPC1) and cold shock domain-containing protein E1 (CSDE1), were detected. Consistent with these results, previous studies in mice have found PABPC1 to be up-regulated during liver regeneration [10]. Similarly, another stress-induced RNA processing protein, the heat shock cognate 71 kDa protein (HSPA8), was found to play a role during rat skeletal muscle regeneration and zebrafish caudal fin regeneration [11,12].

Basic leucine zipper and W2 domain-containing protein 1 (BZW1), protein arginine N-methyltransferase 5 (PRMT5), and SWI/SNF-related matrix-associated actin-dependent regulator of chromatin subfamily A member 5 (SMARCA5) are known for regulation of gene expression. Interestingly, BZW1 has been previously found to induce histone H4 gene expression, which aids the progression of the G1/S phase of the cell cycle [13]. In addition, PRMT5 has been shown to play role in cell proliferation in planaria and be up-regulated post-injury in the kidneys [14,15]. Methionine aminopeptidase 1 (METAP1) and receptor-type tyrosine-protein phosphatase C (PTPRC), also known as CD45, have been shown to play a role in cell activation, proliferation, and cell cycle progression [16-18]. The protein expression data implicate regulation of gene expression as an important event for transdifferentiation.

b. ROS balance

Heme oxygenase 1 (HMOX1), redox-regulatory protein FAM213A, cis-aconitate decarboxylase (IRG1), and serpin B10 (SERPINB10) are known for their association with reactive oxygen species (ROS) and redox balance. IRG1 is activated by ROS to prevent infections [19]. SERPINB10 plays a role in protein processing and it is sensitive to redox stress [20]. HMOX1 is induced by ROS and has been linked to wound healing and regeneration in many regeneration model systems with the exception of mouse liver regeneration [21-24]. ROS stress and redox balance has gained a lot of attention since many studies have associated changes related to them with regenerative responses [25]. Our data indicate a potential role of ROS during newt lens regeneration as well.

c. Immune response

Argininosuccinate synthase (ASS1), complement factor B (CFB), acidic mammalian chitinase (CHIA), bis (5′-adenosyl)-triphosphatase ENPP4, eosinophil peroxidase (EPX), coagulation factor XIII A chain (F13A1), and myeloperoxidase (MPO) are up-regulated during lens regeneration and are known for their roles in preventing infections and generally to facilitate host defense and immune response.

d. Metabolic processes

Sterol O-acyltransferase 1 (SOAT1), delta-1-pyrroline-5-carboxylate synthase (ALDH18A1), v-type proton ATPase subunit d 1 (ATP6V0D1), cytochrome b-245 heavy chain (CYBB), n-acylethanolamine-hydrolyzing acid amidase (NAAA), putative neutrophil cytosol factor 1C (NCF1C), neutrophil cytosol factor 2 (NCF2), and 6-phosphogluconate dehydrogenase, decarboxylating (PGD) participate in many metabolic processes and regulation of energy production. SOAT1 is also known to be involved during rat adrenal regeneration [26].

e. Other functions

Additional interesting proteins, potentially involved in the regulation of newt lens regeneration, are msx2-interacting protein (SPEN), which aids wound healing in *Drosophila* embryos [27]; cathepsin L1 (CTSL1), which mediates proteolysis; DNA topoisomerase 1 (TOP1), which is associated with DNA replication and transcription; epidermal retinol dehydrogenase 2 (SDR16C5), which is involved in making retinoic acid; and ATP-dependent DNA helicase Q1 (RECQL), which mediates DNA repair. Furthermore, we found an up-regulation of unconventional myosin-XVIIIa (MYO18A), which is involved in cell migration, as well as fibronectin (FN1) and integrin beta-2 (ITGB2), which mediate adhesion and cell migration and are essential for zebrafish heart regeneration [28].

The rapid up-regulation of all the aforementioned proteins during lens regeneration clearly underscores the importance of host defense, redox balance, and response to stress for the initiation of regeneration.

### Proteins regulated in dorsal versus ventral iris

Since regeneration only proceeds from the dorsal iris, we wanted to find proteins that were specifically up-regulated in this tissue. To achieve this goal, we compared all proteins up-regulated during lens regeneration to proteins only up-regulated in the dorsal or the ventral iris. Proteins identified were imported in VisANT, a program that analyses protein-protein interactions [29]. Proteins that were expressed at higher levels in dorsal samples represented complexes associated with extracellular matrix, ribosomes, and DNA (Figure 2C) whereas proteins expressed at higher levels in ventral samples did not exhibit such a pattern (Figure 2D).

- Extracellular matrix

Collagen proteins such as COL2A1, COL9A2, and COL9A3 are essential for structural support and provide the substrate for surrounding cells. In addition, COL9A2 and COL9A3 compose the vitreous area of the eye. Versican core protein (VCAN) is known for cell-extracellular matrix interactions allowing cell migration and growth. Laminin subunit alpha-3 (LAMA3) promotes migration. Fibrillin-1 (FBN1), fibrillin-2 (FBN2), and syndecan-2 (SDC2) are extracellular matrix proteins regulating the availability of growth factors to nearby cells. SDC2 also plays a role during rat periodontal wound healing [30]. Annexin A5 (ANXA5) is known for its anticoagulant properties and promotes wound healing in the cornea [31]. Previous studies in newt limb and lens regeneration also support these findings. A study in newt limb regeneration suggests that dynamic changes of the extracellular matrix provide a suitable microenvironment for regeneration [32], which is in-line with the up-regulation of several extracellular matrix genes during newt lens regeneration in the dorsal iris as determined by DNA microarray analysis [8]. These results suggest that remodeling an appropriate environment is a fundamental event during lens regeneration.

b. Cell activation

Rho guanine nucleotide exchange factor 1 (ARHGEF1), ATPase family AAA domain-containing protein 3A (ATAD3A), polypyrimidine tract-binding protein 3 (PTBP3, also known as ROD1), casein kinase I isoform epsilon (CSNK1E), src substrate cortactin (CTTN), replication factor C subunit 2 (RFC2), and NEDD8 have all been shown to have roles in cell proliferation, migration, and growth [33-36]. These cellular events are important since the lost tissue needs to be regenerated.

c. Gene expression

Cullin-5 (CUL5), polyadenylate-binding protein 2 (PABPN1), exosome complex exonuclease RRP44 (DIS3), SHC-transforming protein 1 (SHC1), tyrosine-protein kinase SYK, serine/threonine-protein phosphatase 6 regulatory subunit 1 (PPP6R1), chromodomain-helicase-DNA-binding protein 4 (CHD4), and ribosomal proteins RPL15, RPL32, RPL8, RPL28, RPL21, RPL10, and RPL19 are playing roles in regulating gene expression at various levels [37] and are all up-regulated in the dorsal iris. Interestingly, CHD4 has also been shown to be up-regulated during muscle regeneration in mice [38]. RNA sequencing during lens regeneration has previously revealed that genes associated with the regulation of gene expression are enriched in the dorsal iris, a pattern that we also found here at the protein level [6]. These results highlight the importance of rapid and impactful changes in the regulation of gene expression that will ultimately lead to transdifferentiation of iris cells to lens cells.

d. DNA repair

Poly [ADP-ribose] polymerase 1 (PARP1), DNA-dependent protein kinase catalytic subunit (PRKDC), and structural maintenance of chromosomes protein 1A (SMC1A) have known roles in DNA repair. DNA repair genes such as RAD1 have been previously found to be up-regulated in the dorsal iris using both microarrays and RNA sequencing during newt lens regeneration [6,8]. Such cellular machinery can play a role in maintaining the integrity of the genome an important aspect of regenerating an exact “clone” of the missing lens.

e. Other functions

Other proteins found to be up-regulated in the dorsal iris during lens regeneration were keratin, type II cytoskeletal 6A (KRT6A), and caspase-3 (CASP3) which have been implicated in wound healing and regeneration [39,40].

All these proteins, grouped in the different functional categories, were up-regulated in the dorsal compared to the ventral iris during lens regeneration. Interestingly, several of these proteins formed interacting networks that can be linked to the regeneration process (Figure 2C).

### Validation of changes in expression levels by qPCR

**Figure 3:**
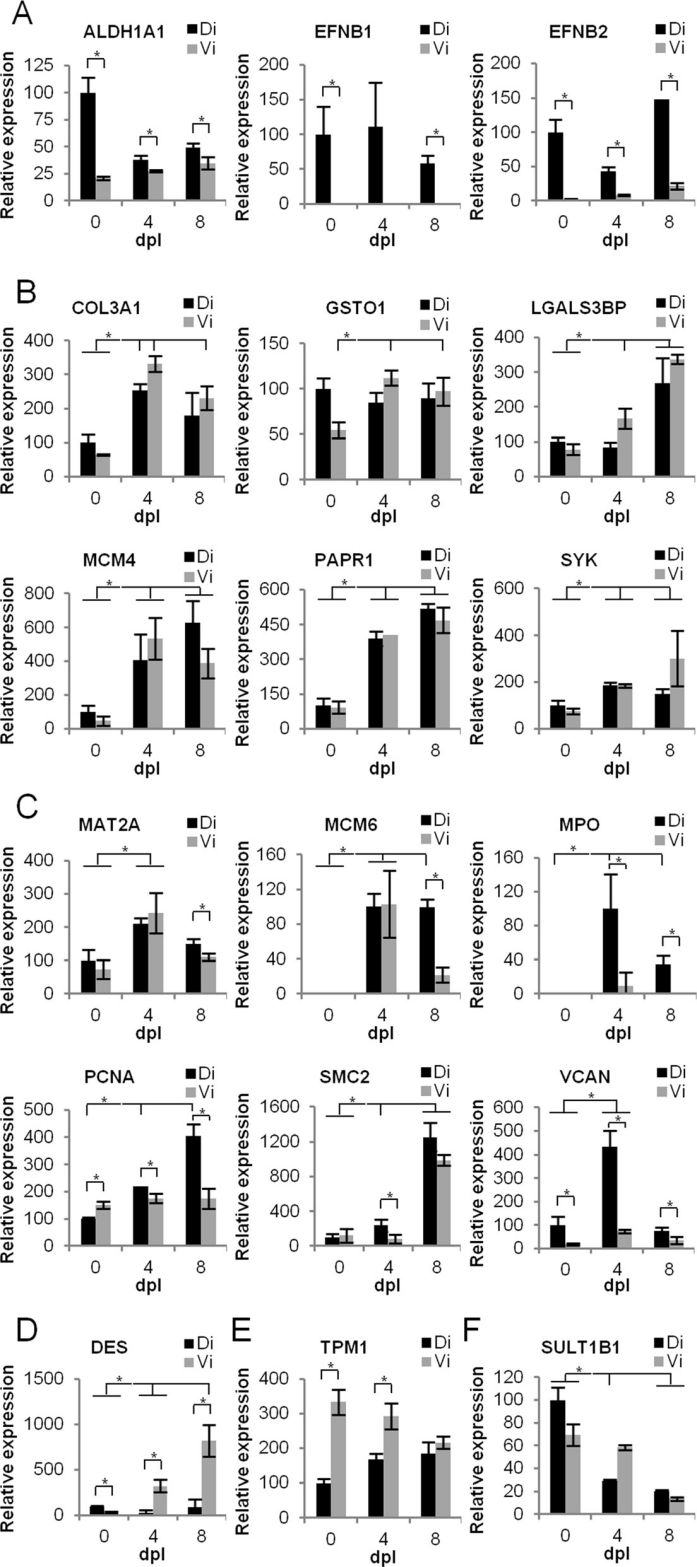
**Validation of protein expression data by qPCR analysis. (A)** Genes expressed at higher levels in dorsal iris. **(B)** Genes expressed at higher levels during regeneration. **(C)** Genes expressed at higher levels in the dorsal iris and during regeneration. **(D)** Gene expressed at higher levels in the ventral iris and during regeneration. **(E)** Gene expressed at higher levels in the ventral iris. **(F)** Gene expressed at higher levels in the intact iris. Student’s *t*-test for independent samples was used for statistical significance. Homoscedasticity was assumed when Levene’s test *p* value was greater than 0.05. *Asterisk* (*) indicates statistical significance (Student’s *t*-test; *p* < 0.05). Each *bar* represents the average of triplicate values. *Error bars* represent standard deviation. *Lines on the top of the graph* compare samples during regeneration and control. *Lines on the top of bars* corresponding to a single day compare dorsal and ventral iris. For simplicity, only the statistics relevant for each group are presented on the graphs.

Since only few newt specific antibodies are available, we used quantitative real-time polymerase chain reaction (qPCR) to validate our data. Although qPCR measures mRNA and not protein concentrations, we reasoned that concomitant changes at the mRNA and protein levels might allow us to validate general patterns of gene activity during regeneration. We selected several proteins based on their putative function. Retinal dehydrogenase 1 (ALDH1A1), ephrin-B1 (EFNB1), and ephrin-B2 (EFNB2) were significantly up-regulated in the dorsal iris compared to the ventral irrespective of the timepoint (*p* < 0.05; Figure 3A). Interestingly, these genes are also expressed during eye development in the dorsal optic cup [41,42] revealing a persisting pattern of gene expression from embryonic development to adulthood in the iris of newts. It is tempting to speculate that such genes may aid or repress regeneration hence providing the intrinsic regeneration potential of the dorsal iris. COL3A1, glutathione S-transferase omega-1 (GSTO1), galectin-3-binding protein (LGALS3BP), DNA replication licensing factor MCM4, PARP1, and SYK were up-regulated during regeneration both in the ventral and the dorsal parts of the iris (*p* < 0.05; Figure 3B). These genes are related to extracellular matrix, cell adhesion, redox balance, DNA maintenance, and DNA repair, processes required for regeneration and wound repair. S-adenosylmethionine synthase isoform type-2 (MAT2A), DNA replication licensing factor MCM6, MPO, proliferating cell nuclear antigen (PCNA), structural maintenance of chromosomes protein 2 (SMC2), and VCAN are also associated with the above-mentioned cellular processes but showed a higher expression in the dorsal versus the ventral iris and were expressed at higher levels during regeneration compared to undamaged controls (*p* < 0.05; Figure 3C) suggesting that the dorsal iris responds more robustly than the ventral iris to regenerative cues. Desmin (DES) is an intermediate filament found mostly in muscle tissue and has been associated with mitochondrial dysfunction and elevated ROS [43]. Desmin is only up-regulated during regeneration in the ventral iris (*p* < 0.05; Figure 3D). Tropomyosin alpha-1 chain (TPM1) is another cytoskeleton protein up-regulated in the ventral iris compared to the dorsal iris (*p* < 0.05; Figure 3E). Lastly, sulfotransferase family cytosolic 1B member 1 (SULT1B1), an enzyme catalyzing sulfonation, was expressed at higher levels in the intact iris (day 0), an expression pattern also found during liver regeneration in rats (Figure 3F) [44]. Overall, the qPCR data corroborated the expression changes found at the proteome level by mass spectrometry.

### Protein expression patterns from the *in vitro* proteome

**Figure 4:**
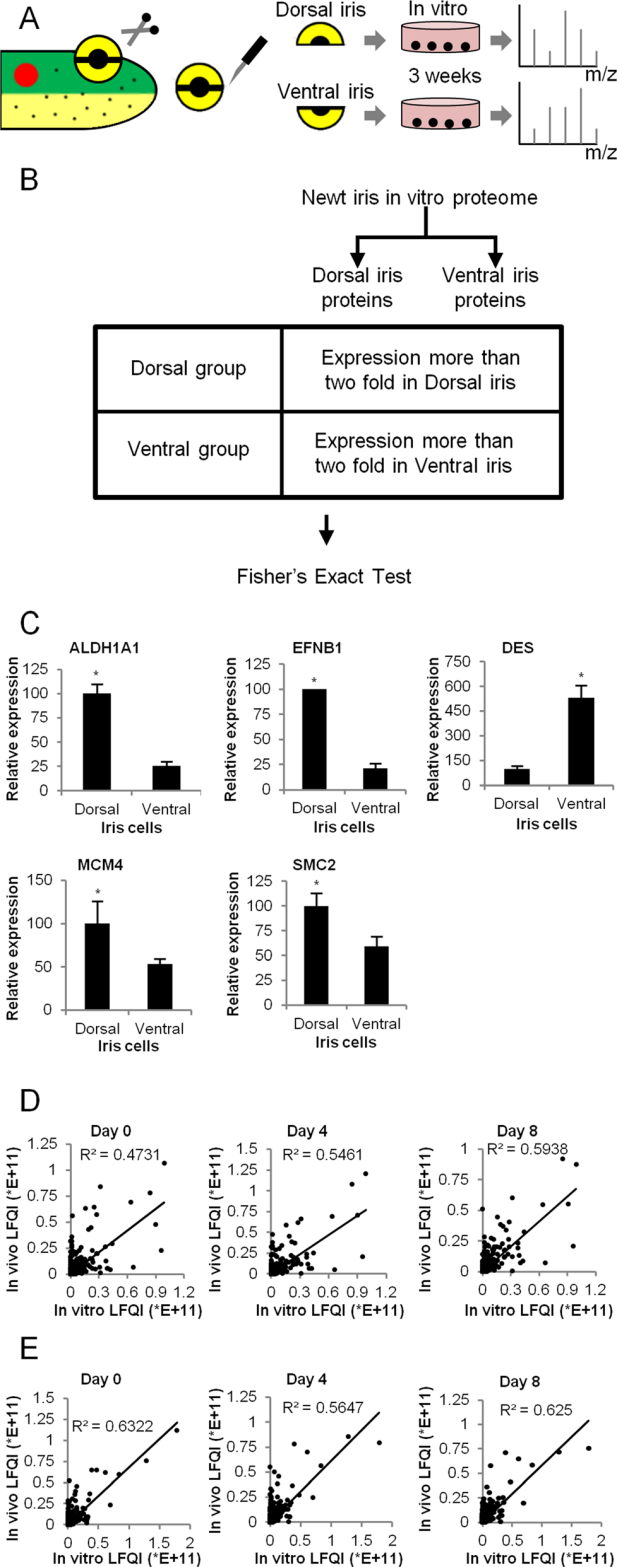
**LC-MS/MS in cultured IPE cells and comparisons with** ***in vivo*** **samples. (A)** Overview of procedure for using LC-MS/MS in cultured iris cells. **(B)** Dorsal and ventral group selection for comparison with Fisher’s exact test. **(C)** qPCR validation of *in vitro* proteomics data and comparisons. Student’s *t*-test for independent samples was used for statistical significance. Equal variances were assumed when Levene’s test *p* value was greater than 0.05. *Asterisk* (*) indicates statistical significance (Student *t*-test; *p* < 0.05). Each *bar* represents the average of triplicate values. *Error bars* represent standard deviation. **(D)** Pearson correlation between expression of dorsal *in vivo* proteins at indicated days and expression of dorsal *in vitro* cultured cell proteins. **(E)** Pearson correlation between expression of proteins *in vivo* in the ventral iris at indicated days and expression of proteins in cultured ventral IPE cells.

IPE cells retain their ability to transdifferentiate to lens cells *in vitro*, a process that can take up to 2 weeks. After re-aggregation and transplantation into a lentectomized eye, only the dorsal but not ventral iris aggregates transdifferentiate to lens cells [45]. Similarly, only dorsal aggregates will transdifferentiate rapidly within 1–2 weeks to a structured lens when placed in 3-D collagen-based lattices like Matrigel [5]. Intriguingly, even IPE cells from higher animals, including humans, can be induced to transdifferentiate into lentoids (not structured lens) under certain culturing conditions [46]. We therefore examined protein expression in cultured IPE cells from the dorsal and ventral iris to monitor potential changes in the state of IPE cells (Figure 4A). In particular, we wanted to know whether culturing changed the protein profile of IPE cells and to identify markers that reflect transdifferentiation.

In total, we identified 2,269 annotated to known human proteins (e-value < 1E-10) in the cultured IPE cells. Proteins showing more than twofold higher expression either in the dorsal or ventral IPE cells are listed in Additional file 2. Numerous proteins were exclusively found either in the dorsal or ventral IPEs although GO terms analysis only revealed enrichment of cytoskeleton-associated terms in ventral versus dorsal IPE cells (Additional file 1 and Figure 4B).

Next, we compared the *in vitro* proteome with the *in vivo* proteome of 0, 4, and 8 dpl. Proteins with a similar expression pattern in respect to the dorsoventral axis under both *in vitro* and *in vivo* conditions are shown in Table 6. Some proteins have cell cycle, DNA replication, and splicing functions in the cell. EFNB1, DES, ALDH1A1, SMC2, and MCM4 proteins showed differences in expression levels between the dorsal and ventral IPE cells, an exact pattern as of their protein expression *in vivo*. As potential dorsoventral markers, we further validated these expression data by qPCR (Figure 4C). EFNB1, ALDH1A1, SMC2, and MCM4 were significantly up-regulated in dorsal IPE cells, while expression of DES was significantly up-regulated in ventral IPE cells (*p* < 0.05; Figure 4C). ALDH1A1, SMC2, and DES, which are similarly regulated in the iris during lens regeneration *in vivo* (Figure 3A,C), are involved in retinoic acid synthesis and DNA replication. Such cellular processes have been previously shown to be involved in lens regeneration from the dorsal iris [6,47]. Pearson correlation analysis of *in vivo* and *in vitro* datasets revealed that the *R*^2^ correlation value increased from 0 to 4 dpl with the highest correlation at 8 dpl, indicating cells activated for tissue regeneration (Figure 4D). In contrast, ventral IPE cells did not show such a correlation or trend (Figure 4E). The expression of dorsal markers ALDH1A1 and EFNB1 in dorsal IPE cells showed that they keep a “memory” of their origin, which consequently might be responsible for their ability to transdifferentiate to lens cells. The identified dorsal- or ventral-specific proteins might be used as markers in high-throughput screening using small molecules to identify agents inducing regeneration.

**Table 6:** **Genes with a similar expression pattern between iris during** ***in vivo*** **lens regeneration and** ***in vitro*** **cultured iris cells**

**Dorsal**							**Ventral**
PHPT1	DDX46	DDX23	CHD4	CDK1	VCAN	SMC2	PLEC
MCM4	ALDH1A1	RANGAP1	APEH	PUS7	P4HA1	GSTO1	DES
PCNA	PARP1	MAT2A	SPTBN1				

### On the road for a common regeneration program

**Figure 5:**
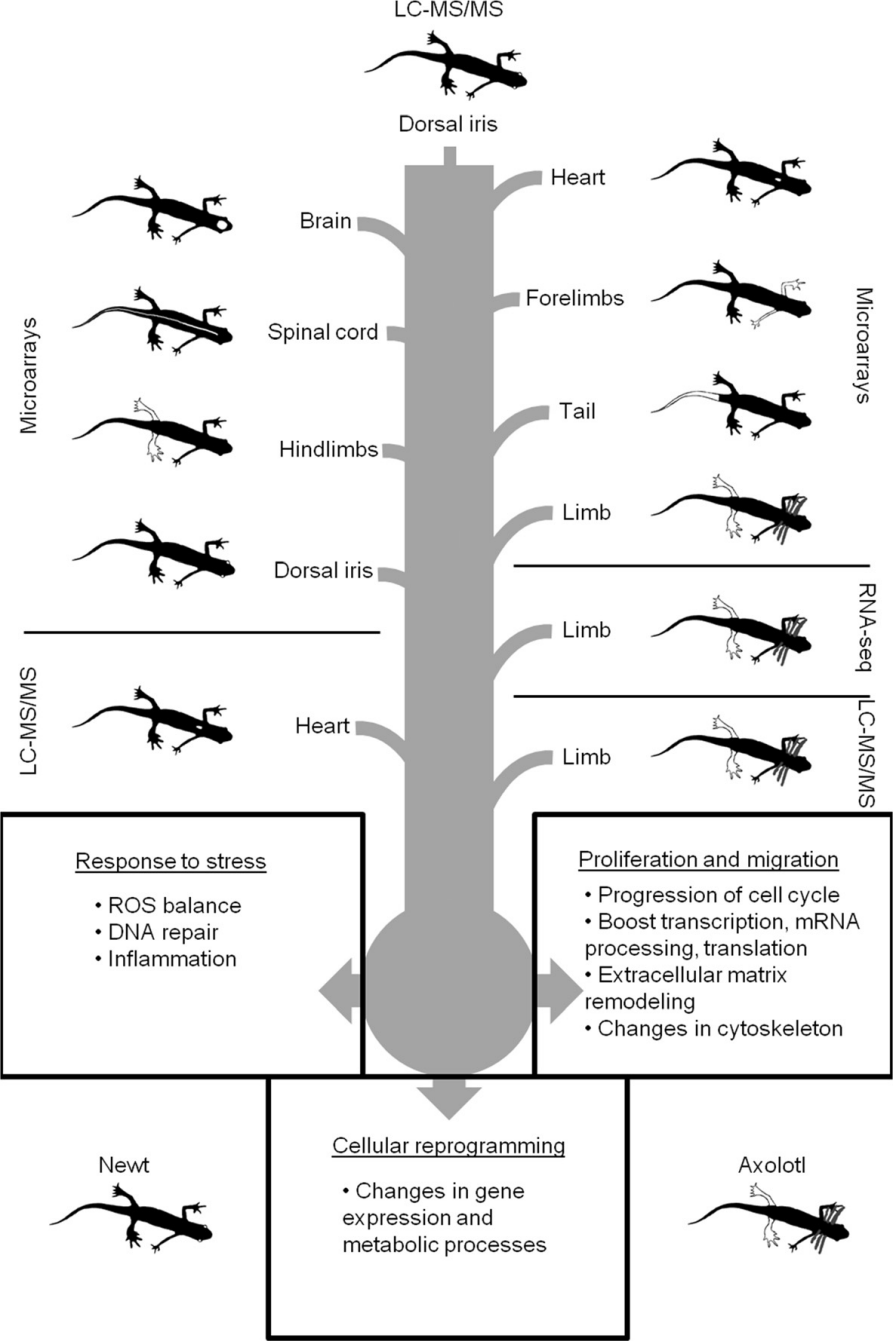
**Gene expression comparison of different regenerating tissues from amphibians reveals a canonical regeneration program.** Proteins found to be up-regulated during regeneration in the dorsal iris in the present study were compared to gene expression datasets related to amphibian regeneration published previously. Datasets included gene expression profiles from DNA microarray analysis of newt brain, spinal cord, hindlimbs, dorsal iris, heart, forelimbs and tail regeneration, microarray and RNA-seq analysis from axolotl limb regeneration, and LC-MS/MS from newt heart and axolotl limb regeneration. Newts and axolotls are presented with *black color*. Respective regenerating organs are colored *white* on the animals. The *central grey column* depicts the collection of all the gene expression data from the different regenerating tissues located at the periphery. The *node* and the *three arrows* represent the result of the data comparison. *Boxes* highlight the three major events of the common canonical regeneration program that was identified.

During the last two decades, several high-throughput methods including microarrays, next-generation RNA sequencing, and mass spectrometry have been developed to characterize gene expression profiles. We have used datasets from several different studies investigating organ regeneration in amphibians and extracted genes that were expressed at higher levels during tissue regeneration compared to intact controls. We focused on genes that were expressed more than twofold at any regeneration timepoint compared to intact tissue (for more information, see the “Methods” section). In addition, we annotated the genes based on human gene names serving as a common reference for the comparisons. Our search included seven microarray datasets from newt brain, spinal cord, hindlimb, forelimb, lens, heart and tail regeneration, one microarray and one RNA-seq dataset from axolotl limb regeneration [48,49], and two LC-MS/MS studies in newt heart regeneration and axolotl hindlimb regeneration [50-52]. We compared these datasets to proteins up-regulated at least twofold in the dorsal iris during lens regeneration compared to intact iris (Figure 5 and Additional file 3). Surprisingly, the highest degree of similarity was found when RNA-seq data from limb regeneration were used (Table 7) [49]. Genes which were jointly activated in these, rather different, tissues during regeneration (Figure 5 and Table 7) most likely represent a part of a canonical regeneration program. Hallmarks of the program include inflammation for host defense and cell activation, proliferation of new cells to replace lost tissue, migration for rearrangement of cells, generation of an appropriate extracellular matrix, regulation of ROS and DNA repair for tissue homeostasis, metabolic processes for cells to meet the needs of energy-consuming cellular processes, and changes in gene expression to shape the newly formed organ. We assume that these functional groups play a decisive role in the majority of all regeneration events.

**Table 7:** **Genes up-regulated during newt lens and axolotl limb regeneration**

ABCA1^1^	C3^3^	EEF1E1^2^	HMOX1^3^	LSP1^3^	NCF2^1^	ROBO1^4^	TGFB1^2^
ABR^2^	CASP3^3^	EMILIN1^4^	HSPA5^3^	MARCO^3^	OPTC^4^	SACS^3^	TGM1^2^
ACSBG2^1^	CFB^3^	ERO1L^3^	HSPA9^3^	MCM4^5^	PAK2^5^	SAMD9L^5^	TGM2^2^
ACSL4^1^	CHIT1^3^	ETF1^2^	IDE^2^	MCM5^5^	PGD^1^	SERPINB10^2^	TGM6^2^
AIF1^3^	CLPTM1^3^	F13A1^3^	IFIH1^3^	MCM6^5^	PLEC^4^	SLC30A1^1^	TLR2^3^
ALOX5^1^	CPT1A^1^	FBLN1^4^	ITGAD^4^	MCM7^5^	PPID^3^	SOAT1^1^	TMEM43^6^
AQR^2^	CSE1L^5^	FBP1^1^	KIAA1967^5^	MOV10^2^	PSEN1^2^	TARS^2^	TNPO3^2^
ASTL^4^	CUL1^5^	FREM2^4^	LAMA3	MPO^3^	PTCD3^2^	TCN1^1^	VAMP8^2^
BCL2L1^5^	CYBA^1^	GIMAP7^2^	LGALS9^3^	MYO1F^6^	PTPRC^5^	TEX2^1^	VCAN^4^
BCS1L^1^	CYBB^1^	HK2^1^	LMNA^4^	NCCRP1^5^	RAI14^6^	TFRC^1^	VSIG4^3^
C1orf85^2^	DNAJC5^1^						

Gene function related to: metabolic process and transporters^1^, gene expression and protein homeostasis^2^, response to stress, host defense, immune response and reactive oxygen species^3^, migration, adhesion and extracellular matrix^4^, cell cycle, cell proliferation and DNA replication^5^, cytoskeleton, cell shape, and organelle shape^6^.

## Conclusions

In this study, we employed LC-MS/MS to identify proteins that are highly expressed during newt lens regeneration. Some of these proteins have similar functions and are arranged in protein networks associated with regulation of the extracellular matrix, DNA repair and maintenance, gene expression, and regulation of translation. Comparisons to other datasets collected during regeneration of a variety of different tissues from amphibians species revealed a putative canonical regeneration program, which seems to be required for regeneration to occur. Finally, we showed that cultured dorsal IPE cells *in vitro* maintain a molecular memory of their origin and show similar patterns as the 8-dpl *in vivo* lens regeneration dorsal iris. Taken together, our study provides information about proteins and protein groups that play an important role during tissue regeneration and deepens our understanding of the mechanism of regeneration.

## Methods

### Animal procedures

Animal handling and operations have been described previously [6,45]. Adult newts, *Notophthalmus viridescens*, were purchased from the Charles Sullivan Inc. Newt Farm. Anesthesia was performed with 0.1%(*w*/*v*) ethyl-3-aminobenzoate methanesulfonic acid (MS222; Sigma-Aldrich, St. Louis, MO) in phosphate buffered saline (PBS; 37 mM NaH_2_PO_4_ monohydrate, 58 mM Na_2_HPO_4_ anhydrous, pH 7.0). All procedures involving animals were approved by the University of Dayton Institutional Animal care and Use Committee.

### Sample collection for LC-MS/MS

Newts were anesthetized and whole eye balls were removed and placed in calcium- and magnesium-free (CMF) Hank’s solution. Using scissors, eye balls were dissected and iris rings were isolated. Using a scalpel, dorsal and ventral 135° sectors were extracted and kept frozen at −70°C until use.

### Sample collection for qPCR, RNA extraction, reverse transcriptase reaction, qPCR reactions, and enrichment analysis

All procedures were performed as described previously [6]. For primers and quantitative real-time polymerase chain reaction (qPCR) settings see Additional file 4. Student’s *t*-test for independent samples was used to determine statistical significance for qPCR expression data. Equal variances were determined with Levene’s test. Groups for enrichment analysis were selected as follows: For *in vivo* proteome analysis, protein expression was detected at 0 dpl and at least for one of the 4- and 8-dpl samples. Only differences more than twofold were used for further bioinformatical analysis. Annotation was assigned to newt proteins using BLAST2GO with e-value less than 1E-10. GO mapping and annotation was performed with default settings. GO enrichment analysis was calculated using Fisher’s exact test corrected for multiple selections which is a built-in feature of BLAST2GO [53,54]. GO terms were considered enriched when FDR < 0.05.

### Network analysis

Network analysis was performed using VisANT [29]. For construction of the dorsal regeneration network, only proteins with more than twofold change during regeneration (4 and/or 8 dpl) compared to the intact control and more than twofold change compared to the equivalent timepoints in the ventral iris samples were included. The selected proteins were used as input for the program. Human gene names and the human interactome were used for this analysis. Only proteins that had at least one interaction with a different protein of the group were displayed. The same procedure was used for the construction of the ventral regeneration network.

### Newt IPE cell culture

Newt dorsal and ventral IPE cell culture was performed as described previously with minor modifications [45]. Dorsal and ventral IPE cells were plated separately in DMEM on collagen I coated plates (Becton Dickinson, Franklin Lakes, NJ). Cells were incubated at 27°C with 2% CO_2_. The medium was changed every other day till day 21. On day 21, dispase (Gibco, Life technologies, Grand Island, NY) was added to the medium with a final concentration of 5% (*v*/*v*) and incubated overnight at 27°C with 2% CO_2_. The collected cells were washed thrice with CMF Hank’s solution and frozen with liquid nitrogen until use.

### LC-MS/MS procedures

The iris tissue and cultured cells were isolated as described above. Proteins were isolated as described in [55] and processed for mass spectrometry (reverse-phase nano-LC-MS/MS, Thermo Velos and Q Exactive, Thermo Scientific, Waltham, MA) measurements. In brief, proteins were isolated by homogenizing tissue/cells in a buffer containing 1% Nonidet P-40, 0.1% sodium deoxycholate, 150 mm NaCl, 1 mm EDTA, and 50 mm Tris, pH 7.5 and protease inhibitor mixture (Roche, San Francisco, CA). Next, proteins were separated by 1D SDS-PAGE and stained by Coomassie Blue. The gel was cut into eight slices per lane (each timepoint *in vivo*, dorsal and ventral *in vitro*). Gel slices were washed by 100 μL 50 mM ammonium bicarbonate (ABC)/50% ethanol (EtOH) for 20 min at RT and dehydrated by incubating for 10 min in 100 μL absolute EtOH. Protein reduction was performed by incubating the gel pieces in 100 μL 10 mM DTT (in 50 mM ABC) for 45 min at 56°C. Alkylation was done by incubating the gel pieces in 100 μL 55 mM iodacetamide for 30 min at RT in darkness. After a final washing step, gel pieces were dried and proteins were in-gel digested using trypsin overnight. For desalting, peptides were loaded onto STAGE-tips and eluted with 80% acetonitril for mass spectrometry (MS) analysis [56,57]. Reversed-phase nano-LC-MS/MS was performed by using an Easy nanoflow HPLC system (Thermo Fisher Scientific, Odense Denmark; binary buffer system of A (0.1% (*v*/*v*) formic acid in H20) and B (0.1% (*v*/*v*) formic acid in 80% acetonitrile); 50-cm column (75-μm ID) packed in-house with 1.9-μm diameter C18 resin). The HPLC is coupled to Q Exactive mass spectrometer (Thermo Fisher Scientific, Bremen, Germany) with an electrospray ionization source (Thermo Fisher Scientific, Bremen, Germany). MS spectra were acquired at a resolution of 70,000 (200 m/z) in a mass range of 350–1,650 m/z and the top 10 most intense ions were selected for fragmentation. To identify mass-spectrometry-derived spectra, a *de-novo-*assembled transcriptome [7] was utilized by translating it into six reading frames generating an Andromeda search engine [58] compatible database. Only reading frames greater than 50 AA were used. Subsequent protein identification and label-free quantification was performed using MaxQuant software (Version 1.2.0.18) [59]. The maximum false discovery rate was set below 1% for peptide and protein identifications using the DECOY target database approach. Minimum peptide length was set to 7 AA and two peptides per protein group (at least one unique peptide). Carbamidomethyl at cysteine residues was set as a fixed modification. Oxidation at methionine and acetylation at the N-terminus were defined as variable modifications. Label-free quantification was based on at least two ratio counts. *In vivo* and *in vitro* LC-MS/MS data can be found in Additional file 2.

### Comparisons with other datasets

Microarray, RNA sequencing, and LC-MS/MS data were extracted from the following papers: newt heart [50], forelimb, hindlimb, spinal cord, tail, brain, heart, tail [51], lens (dorsal iris) [8], and axolotl limb regeneration [48,49,52]. Genes were selected based on two expression criteria: expressed more than twofold in any of the regeneration timepoints compared to the control and not expressed more than twofold in the control compared to any of the regeneration timepoints. Human gene names were assigned to the extracted genes from all the datasets based on the annotation provided in the corresponding papers. Ambiguous annotations were discarded. The extracted genes can be found in Additional file 3. Comparisons, annotation assignments, and data mining were performed using custom Perl scripts.

### Comparison between *in vitro* LC-MS/MS and *in vivo* LC-MS/MS data

*In vitro* LC-MS/MS data and *in vivo* LC-MS/MS data were normalized together for better correlation of expression levels. Pearson correlation was performed between dorsal IPE cell protein expression and the different timepoints of *in vivo* dorsal iris. Similar comparisons were performed with ventral samples. Tests were performed using Microsoft Office Excel spreadsheets.

## Additional files

Additional file 1:
**Gene ontology enrichment analysis.** Enrichment analysis using Fisher’s exact test for genes up-regulated during *in vivo* regeneration versus control and *in vitro* ventral versus dorsal IPE cells. Additional file 2:
**Protein expression data.** Protein expression data from LC-MS/MS for 0, 4, and 8 dpl dorsal and ventral iris, dorsal and ventral IPE *in vitro* cultured cells, and genes up-regulated at least twofold in dorsal or ventral IPE cells. Additional file 3:
**Gene pools for common regeneration program analysis.** Genes from previous high-throughput amphibian studies, which were up-regulated during different timepoints of regenerating versus control. Additional file 4:
**Primer sequences and qPCR/PCR settings.** All primers and qPCR/PCR settings that were used for this study.

## References

[1] Baddour JA, Sousounis K, Tsonis PA (2012). Organ repair and regeneration: an overview. Birth Defects Res C Embryo Today.

[2] Barbosa-Sabanero K, Hoffmann A, Judge C, Lightcap N, Tsonis PA, Del Rio-Tsonis K (2012). Lens and retina regeneration: new perspectives from model organisms. Biochem J.

[3] Eguchi G, Shingai R (1971). Cellular analysis on localization of lens forming potency in the newt iris epithelium. Dev Growth Differ.

[4] Wolff G (1895). Entwickelungsphysiologische studien. I. Die regeneration der urodelenlinse. Arch EntwMech Org.

[5] Hoffmann A, Nakamura K, Tsonis PA (2014). Intrinsic lens forming potential of mouse lens epithelial versus newt iris pigment epithelial cells in three-dimensional culture. Tissue Eng Part C Methods.

[6] Sousounis K, Looso M, Maki N, Ivester CJ, Braun T, Tsonis PA (2013). Transcriptome analysis of newt lens regeneration reveals distinct gradients in gene expression patterns. PLoS One.

[7] Looso M, Preussner J, Sousounis K, Bruckskotten M, Michel CS, Lignelli E, Reinhardt R, Hoeffner S, Krueger M, Tsonis PA, Borchardt T, Braun T (2013). A *de novo* assembly of the newt transcriptome combined with proteomic validation identifies new protein families expressed during tissue regeneration. Genome Biol.

[8] Sousounis K, Michel CS, Bruckskotten M, Maki N, Borchardt T, Braun T, Looso M, Tsonis PA (2013). A microarray analysis of gene expression patterns during early phases of newt lens regeneration. Mol Vis.

[9] Frye BC, Halfter S, Djudjaj S, Muehlenberg P, Weber S, Raffetseder U, En-Nia A, Knott H, Baron JM, Dooley S, Bernhagen J, Mertens PR (2009). Y-box protein-1 is actively secreted through a non-classical pathway and acts as an extracellular mitogen. EMBO Rep.

[10] Hsieh HC, Chen YT, Li JM, Chou TY, Chang MF, Huang SC, Tseng TL, Liu CC, Chen SF (2009). Protein profilings in mouse liver regeneration after partial hepatectomy using iTRAQ technology. J Proteome Res.

[11] Duguez S, Bihan MC, Gouttefangeas D, Feasson L, Freyssenet D (2003). Myogenic and nonmyogenic cells differentially express proteinases, Hsc/Hsp70, and BAG-1 during skeletal muscle regeneration. Am J Physiol Endocrinol Metab.

[12] Tawk M, Joulie C, Vriz S (2000). Zebrafish Hsp40 and Hsc70 genes are both induced during caudal fin regeneration. Mech Dev.

[13] Mitra P, Vaughan PS, Stein JL, Stein GS, van Wijnen AJ (2001). Purification and functional analysis of a novel leucine-zipper/nucleotide-fold protein, BZAP45, stimulating cell cycle regulated histone H4 gene transcription. Biochemistry.

[14] Braun MC, Kelly CN, Prada AE, Mishra J, Chand D, Devarajan P, Zahedi K (2004). Human PRMT5 expression is enhanced during *in vitro* tubule formation and after *in vivo* ischemic injury in renal epithelial cells. Am J Nephrol.

[15] Rouhana L, Vieira AP, Roberts-Galbraith RH, Newmark PA (2012). PRMT5 and the role of symmetrical dimethylarginine in chromatoid bodies of planarian stem cells. Development.

[16] Morikawa K, Oseko F, Morikawa S (1991). The role of CD45 in the activation, proliferation and differentiation of human B lymphocytes. Int J Hematol.

[17] Shivtiel S, Lapid K, Kalchenko V, Avigdor A, Goichberg P, Kalinkovich A, Nagler A, Kollet O, Lapidot T (2011). CD45 regulates homing and engraftment of immature normal and leukemic human cells in transplanted immunodeficient mice. Exp Hematol.

[18] Hu X, Addlagatta A, Lu J, Matthews BW, Liu JO (2006). Elucidation of the function of type 1 human methionine aminopeptidase during cell cycle progression. Proc Natl Acad Sci U S A.

[19] Li Y, Zhang P, Wang C, Han C, Meng J, Liu X, Xu S, Li N, Wang Q, Shi X, Cao X (2013). Immune responsive gene 1 (IRG1) promotes endotoxin tolerance by increasing A20 expression in macrophages through reactive oxygen species. J Biol Chem.

[20] Przygodzka P, Ramstedt B, Tengel T, Larsson G, Wilczynska M (2010). Bomapin is a redox-sensitive nuclear serpin that affects responsiveness of myeloid progenitor cells to growth environment. BMC Cell Biol.

[21] Ueno S, Campbell J, Fausto N (2006). Reactive oxygen species derived from NADPH oxidase system is not essential for liver regeneration after partial hepatectomy. J Surg Res.

[22] Lyoumi S, Puy H, Tamion F, Scotte M, Daveau M, Nordmann Y, Lebreton JP, Deybach JC (1998). Nitric oxide synthase inhibition and the induction of cytochrome P-450 affect heme oxygenase-1 messenger RNA expression after partial hepatectomy and acute inflammation in rats. Crit Care Med.

[23] Nada SE, Tulsulkar J, Shah ZA (2013). Heme oxygenase 1-mediated neurogenesis is enhanced by Ginkgo biloba (EGb 761(R)) after permanent ischemic stroke in mice. Mol Neurobiol.

[24] Wagener FA, van Beurden HE, von den Hoff JW, Adema GJ, Figdor CG (2003). The heme-heme oxygenase system: a molecular switch in wound healing. Blood.

[25] Love NR, Chen Y, Ishibashi S, Kritsiligkou P, Lea R, Koh Y, Gallop JL, Dorey K, Amaya E (2013). Amputation-induced reactive oxygen species are required for successful Xenopus tadpole tail regeneration. Nat Cell Biol.

[26] Tyczewska M, Rucinski M, Ziolkowska A, Trejter M, Szyszka M, Malendowicz LK (2014). Expression of selected genes involved in steroidogenesis in the course of enucleation-induced rat adrenal regeneration. Int J Mol Med.

[27] Mace KA, Pearson JC, McGinnis W (2005). An epidermal barrier wound repair pathway in *Drosophila* is mediated by grainy head. Science.

[28] Wang J, Karra R, Dickson AL, Poss KD (2013). Fibronectin is deposited by injury-activated epicardial cells and is necessary for zebrafish heart regeneration. Dev Biol.

[29] Hu Z, Snitkin ES, DeLisi C (2008). VisANT: an integrative framework for networks in systems biology. Brief Bioinform.

[30] Worapamorn W, Xiao Y, Li H, Young WG, Bartold PM (2002). Differential expression and distribution of syndecan-1 and −2 in periodontal wound healing of the rat. J Periodontal Res.

[31] Watanabe M, Kondo S, Mizuno K, Yano W, Nakao H, Hattori Y, Kimura K, Nishida T (2006). Promotion of corneal epithelial wound healing *in vitro* and *in vivo* by annexin A5. Invest Ophthalmol Vis Sci.

[32] Calve S, Odelberg SJ, Simon HG (2010). A transitional extracellular matrix instructs cell behavior during muscle regeneration. Dev Biol.

[33] Gilquin B, Cannon BR, Hubstenberger A, Moulouel B, Falk E, Merle N, Assard N, Kieffer S, Rousseau D, Wilder PT, Weber DJ, Baudier J (2010). The calcium-dependent interaction between S100B and the mitochondrial AAA ATPase ATAD3A and the role of this complex in the cytoplasmic processing of ATAD3A. Mol Cell Biol.

[34] Sadvakassova G, Dobocan MC, Difalco MR, Congote LF (2009). Regulator of differentiation 1 (ROD1) binds to the amphipathic C-terminal peptide of thrombospondin-4 and is involved in its mitogenic activity. J Cell Physiol.

[35] Tano K, Mizuno R, Okada T, Rakwal R, Shibato J, Masuo Y, Ijiri K, Akimitsu N (2010). MALAT-1 enhances cell motility of lung adenocarcinoma cells by influencing the expression of motility-related genes. FEBS Lett.

[36] Daub H, Olsen JV, Bairlein M, Gnad F, Oppermann FS, Korner R, Greff Z, Keri G, Stemmann O, Mann M (2008). Kinase-selective enrichment enables quantitative phosphoproteomics of the kinome across the cell cycle. Mol Cell.

[37] Querido E, Blanchette P, Yan Q, Kamura T, Morrison M, Boivin D, Kaelin WG, Conaway RC, Conaway JW, Branton PE (2001). Degradation of p53 by adenovirus E4orf6 and E1B55K proteins occurs via a novel mechanism involving a Cullin-containing complex. Genes Dev.

[38] Mammen AL, Casciola-Rosen LA, Hall JC, Christopher-Stine L, Corse AM, Rosen A (2009). Expression of the dermatomyositis autoantigen Mi-2 in regenerating muscle. Arthritis Rheum.

[39] Boland K, Flanagan L, Prehn JH (2013). Paracrine control of tissue regeneration and cell proliferation by Caspase-3. Cell Death Dis.

[40] Pechter PM, Gil J, Valdes J, Tomic-Canic M, Pastar I, Stojadinovic O, Kirsner RS, Davis SC (2012). Keratin dressings speed epithelialization of deep partial-thickness wounds. Wound Repair Regen.

[41] Fan X, Molotkov A, Manabe S, Donmoyer CM, Deltour L, Foglio MH, Cuenca AE, Blaner WS, Lipton SA, Duester G (2003). Targeted disruption of Aldh1a1 (Raldh1) provides evidence for a complex mechanism of retinoic acid synthesis in the developing retina. Mol Cell Biol.

[42] Triplett JW, Feldheim DA (2012). Eph and ephrin signaling in the formation of topographic maps. Semin Cell Dev Biol.

[43] Maloyan A, Osinska H, Lammerding J, Lee RT, Cingolani OH, Kass DA, Lorenz JN, Robbins J (2009). Biochemical and mechanical dysfunction in a mouse model of desmin-related myopathy. Circ Res.

[44] Dunn RT, Kolaja KL, Klaassen CD (1999). Effect of partial hepatectomy on the expression of seven rat sulphotransferase mRNAs. Xenobiotica.

[45] Bhavsar RB, Nakamura K, Tsonis PA (2011). A system for culturing iris pigment epithelial cells to study lens regeneration in newt. J Vis Exp.

[46] Tsonis PA, Jang W, Del Rio-Tsonis K, Eguchi G (2001). A unique aged human retinal pigmented epithelial cell line useful for studying lens differentiation *in vitro*. Int J Dev Biol.

[47] Tsonis PA, Trombley MT, Rowland T, Chandraratna RA, del Rio-Tsonis K (2000). Role of retinoic acid in lens regeneration. Dev Dyn.

[48] Campbell LJ, Suarez-Castillo EC, Ortiz-Zuazaga H, Knapp D, Tanaka EM, Crews CM (2011). Gene expression profile of the regeneration epithelium during axolotl limb regeneration. Dev Dyn.

[49] Stewart R, Rascon CA, Tian S, Nie J, Barry C, Chu LF, Ardalani H, Wagner RJ, Probasco MD, Bolin JM, Leng N, Sengupta S, Volkmer M, Habermann B, Tanaka EM, Thomson JA, Dewey CN (2013). Comparative RNA-seq analysis in the unsequenced axolotl: the oncogene burst highlights early gene expression in the blastema. PLoS Comput Biol.

[50] Looso M, Michel CS, Konzer A, Bruckskotten M, Borchardt T, Kruger M, Braun T (2012). Spiked-in pulsed *in vivo* labeling identifies a new member of the CCN family in regenerating newt hearts. J Proteome Res.

[51] Mercer SE, Cheng CH, Atkinson DL, Krcmery J, Guzman CE, Kent DT, Zukor K, Marx KA, Odelberg SJ, Simon HG (2012). Multi-tissue microarray analysis identifies a molecular signature of regeneration. PLoS One.

[52] Rao N, Jhamb D, Milner DJ, Li B, Song F, Wang M, Voss SR, Palakal M, King MW, Saranjami B, Nye HL, Cameron JA, Stocum DL (2009). Proteomic analysis of blastema formation in regenerating axolotl limbs. BMC Biol.

[53] Conesa A, Gotz S, Garcia-Gomez JM, Terol J, Talon M, Robles M (2005). Blast2GO: a universal tool for annotation, visualization and analysis in functional genomics research. Bioinformatics.

[54] Myhre S, Tveit H, Mollestad T, Laegreid A (2006). Additional gene ontology structure for improved biological reasoning. Bioinformatics.

[55] Looso M, Borchardt T, Kruger M, Braun T (2010). Advanced identification of proteins in uncharacterized proteomes by pulsed *in vivo* stable isotope labeling-based mass spectrometry. Mol Cell Proteomics.

[56] Shevchenko A, Wilm M, Vorm O, Mann M (1996). Mass spectrometric sequencing of proteins silver-stained polyacrylamide gels. Anal Chem.

[57] Andersen JS, Lam YW, Leung AK, Ong SE, Lyon CE, Lamond AI, Mann M (2005). Nucleolar proteome dynamics. Nature.

[58] Cox J, Neuhauser N, Michalski A, Scheltema RA, Olsen JV, Mann M (2011). Andromeda: a peptide search engine integrated into the MaxQuant environment. J Proteome Res.

[59] Cox J, Mann M (2008). MaxQuant enables high peptide identification rates, individualized p.p.b.-range mass accuracies and proteome-wide protein quantification. Nat Biotechnol.

